# New micro/mesoporous nanocomposite material from low-cost sources for the efficient removal of aromatic and pathogenic pollutants from water

**DOI:** 10.3762/bjnano.10.11

**Published:** 2019-01-09

**Authors:** Emmanuel I Unuabonah, Robert Nöske, Jens Weber, Christina Günter, Andreas Taubert

**Affiliations:** 1 Environmental and Chemical Processes Research Laboratory, Centre for Chemical and Biochemical Research, Redeemer’s University, PMB 230, Ede, Osun State, Nigeria; 2 Department of Chemical Sciences, Redeemer’s University, PMB 230, Ede, Osun State, Nigeria; 3 Institute of Chemistry, University of Potsdam, 14476 Potsdam, Germany; 4 State Key Laboratory of Catalysis, Dalian Institute of Chemical Physics, Chinese Academy of Sciences, Dalian, 116023, China; 5 Department of Chemistry, Hochschule Zittau/Görlitz (University of Applied Science), Theodor-Körner-Allee 16, 02763 Zittau, Germany; 6 Department of Earth and Environmental Science, University of Potsdam, 14476 Potsdam, Germany

**Keywords:** 4-nitrophenol, *Carica papaya* seeds, clay, *E. coli*, micro/mesoporous, nanocomposite, water remediation

## Abstract

A new micro/mesoporous hybrid clay nanocomposite prepared from kaolinite clay, *Carica papaya* seeds, and ZnCl_2_ via calcination in an inert atmosphere is presented. Regardless of the synthesis temperature, the specific surface area of the nanocomposite material is between ≈150 and 300 m^2^/g. The material contains both micro- and mesopores in roughly equal amounts. X-ray diffraction, infrared spectroscopy, and solid-state nuclear magnetic resonance spectroscopy suggest the formation of several new bonds in the materials upon reaction of the precursors, thus confirming the formation of a new hybrid material. Thermogravimetric analysis/differential thermal analysis and elemental analysis confirm the presence of carbonaceous matter. The new composite is stable up to 900 °C and is an efficient adsorbent for the removal of a water micropollutant, 4-nitrophenol, and a pathogen, *E. coli,* from an aqueous medium, suggesting applications in water remediation are feasible.

## Introduction

Porous carbon-based materials and carbon/inorganic hybrid materials have extensively been used for the adsorption of pollutants, such as heavy metals or aromatic hydrocarbons, from water in developing countries [1–4]. The removal of such contaminants is a necessity for public health in many developing countries because these pollutants can negatively alter important biochemical processes and thus are a critical threat to the health of plants, animals, and humans [5]. In addition to chemical contaminants, the removal of biological pollutants from water is a key issue in water treatment and public health. According to the World Health Organization, increasing discharges of untreated sewage, in addition to agricultural runoff and wastewater from industry, have drastically reduced the quality of water around the world. The problem is expected to persist in the coming decades if not quickly addressed [6]. For example, the *Escherichia coli* (*E. coli*) O157:H7 strain causes diarrhea, hemorrhagic colitis, and hemolytic uremic syndrome [7] with serious consequences for the infected individuals.

Unfortunately, many pathogens have developed multiple resistances over the years. Moreover, as bacteria also have a spore or vegetative phase, biological pathogens can persist for a very long time in the environment and withstand common disinfection methods [7–8]. Thus, there is a real need for advanced water treatment processes that overcome these issues.

When water remediation in developing countries is considered, the price of the materials and not their performance is unfortunately the key aspect to take into account. Current technologies such as activated carbon or silica-based materials are still too expensive for these situations. As a result, cheap yet highly efficient materials that can be used for the purification of very large volumes of water are therefore highly sought after [4]. This approach applies to all subgroups of contaminants – heavy metals, organic pollutants, and biological contaminants.

One approach to reduce cost while maintaining the functionality of the material is the use of kaolinite as a cheap yet functional component. Indeed, kaolinite-based materials are efficient materials for the desulphurization of crude oil and heavy metal removal from aqueous media [9–14].

Although effective, the preparation of these materials still often involves metakaolinization, dealumination, ageing, and autoclave heating for several days [15–16]. In some cases, silica, alumina, and structure-directing agents have to be added to the metakaolin to tune the properties of the resulting materials [13–14]. These approaches are less expensive than the use of all-synthetic reagents, such as highly developed silica materials, but they are tedious and still rely on autoclave technology, which is not generally available to the countries in need of these materials. Thus, there is a need for cheap, high volume, and low-tech processes towards materials for water treatment. This is particularly important for developing countries, but with sustainability becoming one of the world’s core issues, interest in such materials and processes has also soared in highly industrialized countries.

Indeed, there are reports of the use of clay/carbon materials for the removal of toxic micropollutants from water. In some cases, sugars have been used as starting compounds [17–18], while in other cases, dyes and other organic molecules have been loaded into the clay interlayer and calcined together with the clay to produce hybrid carbon/inorganic adsorbents [19–20].

We have previously described the synthesis and performance of a new hybrid material based on kaolinite and *Carica papaya* seeds for water treatment [1]. The material has several advantages over conventional processes: (i) it can be made from local raw materials, (ii) the raw materials are either waste or very cheap, (iii) the process does not require a complex technical setup, (iv) the process can be adapted to local requirements, such as exchanging the papaya seeds with other organic components that are available locally, and (v) the material performs very well (according to the World Health Organization limits) for Cd(II) and Pb(II) removal from water. However, recent experiments in our laboratories (unpublished) show that the material performs very poorly when attempting to remove anionic pollutants from water.

In response to this observation, we have modified the synthesis strategy using microwave irradiation rather than thermal treatment during synthesis. The resulting materials have been successfully used for the removal of phosphates and gram-negative bacteria from aqueous media [21–22].

Unfortunately, these materials are not effective in the removal of nitrophenol (anionic) pollutants from water. The materials are only able to disinfect water by adsorption without killing the pathogens [22]. The current study shows that these limitations are, however, surmounted by a further modification of the base materials via an improved materials synthesis procedure.

The current study describes the synthesis of a new porous nanocomposite material. Unlike earlier versions of these materials prepared with alkali activation [1,22], the resulting micro/mesoporous carbon–clay nanocomposite in this study shows a high efficiency for the removal of anionic organic and pathogenic pollutants from water. As in the previous study [1], the starting materials are purified raw kaolinite clay (one of the most abundant materials on earth), *Carica papaya* seeds, and ZnCl_2_. The synthesis protocol is very simple and thus is amenable to upscaling and fabrication in less developed regions of the world. No high pressure nor additional (expensive) and environmentally harmful template is necessary.

## Materials and Methods

### Materials

ZnCl_2_, MgCl_2_ (≥98%) and HNO_3_ (>90%) were purchased from Sigma-Aldrich. *Carica papaya* seeds were obtained from the local market in Benin City, Nigeria and sun-dried until all fleshy parts of the fruits were dried off the seeds. The dry seeds were collected into an airtight container. Kaolinite clay was collected from Redeemer's University, Redemption City, Nigeria, and purified according to the method described by Adebowale et al. [23].

### Methods

**Synthesis*.*** Hybrid clay (HYCA) materials were prepared by vigorously mixing MgCl_2_ or ZnCl_2_, kaolinite clay, and papaya seeds (in specific ratios as described in the section “Sample nomenclature” below, 10 g of material in total) in 100 mL of Millipore water in a beaker yielding a turbid paste. These pastes were allowed to stand open to the air for 24 h, after which they were dried in an oven at 70 °C. The dried samples were heated to between 450 and 900 °C in a Carbolite furnace in N_2_ for 2 h. Subsequently, the samples were washed with 3 M HNO_3_ for 30 min with intermittent stirring, filtered, and dried in an oven at 70 °C.

**Sample nomenclature.** The samples prepared with ZnCl_2_ in a 1:1:1 (ZnCl_2_:papaya seeds:kaolinite clay, 10 g total mass) weight ratio were labelled 1Z-HYCA, and samples with a 2:1:1 weight ratio were labelled 2Z-HYCA. Samples prepared with MgCl_2_ were labelled following the same concept but using the prefix M instead of Z. The total mass of all components combined was always 10 g.

### Physicochemical characterization

#### X-ray powder diffraction analysis

The X-ray diffraction analysis data were collected on a PANalytical Empyrean powder X-ray diffractometer in a Bragg–Brentano geometry. This instrument was equipped with a PIXcel1D detector using Cu Kα radiation (λ = 1.5419 Å) operating at 40 kV and 40 mA. The θ/θ scans were run in a 2θ range of 4–70° with a step size of 0.0131° and a sample rotation time of 1 s. The diffractometer was equipped with a programmable divergence and antiscatter slit and a large Nibeta filter. The detector was set to continuous mode with an active length of 3.0061°.

#### Scanning electron microscopy (SEM) and specific surface area analysis

SEM analysis was performed on a Japan Electron Optics Laboratory JSM 6510 with an energy dispersive X-ray spectrometer (Oxford INCAx-act SN detector) to determine the morphology of particles prepared in this study. The porosity analysis and specific surface area determination were performed using Autosorb-1MP and Quadrasorb-MP machines (both Quantachrome Instruments). The samples were degassed under high vacuum at 150 °C for 20 h prior to analysis. The surface areas were calculated either by the single-point or multipoint Brunauer–Emmett–Teller (BET) method [24]. The pore size distributions were calculated using the quenched solid density functional theory (QSDFT) methodology (part of the QuadraWin 5.05 Software package of Quantachrome Instruments). The QSDFT analysis was obtained from the adsorption branch of the isotherms assuming slit-like micropores and cylindrical mesopores.

#### Spectroscopy analysis

Fourier transform infrared spectra (FT-IR) were obtained from transmission measurements (Shimadzu 8400S FTIR, 4000–400 cm^−1^, 40 Scans) using KBr pellets prepared with a Shimadzu MHP-1 mini hand press. The background correction was performed with a pure KBr pellet, and the samples were measured at 10% in 90% KBr. UV–vis spectroscopy was performed on a Shimadzu 1650pc UV–vis spectrophotometer for analysis of 4-nitrophenol. Cross-polarized magic angle spinning nuclear magnetic resonance ^27^Al (104.1 MHz), ^13^C (100.5 MHz), and ^29^Si (79.4 MHz) spectra of the 2Z-HYCA@650 °C nanocomposite were recorded on a Bruker DRX-400 spectrometer with a magic angle spin probe and 4 mm ZrO_2_ rotor ^27^Al signals were referenced to a 0.5 M aqueous solution of aluminum nitrate. ^13^C and ^29^Si signals were referenced to tetramethylsilane (TMS).

#### Other analysis

Thermogravimetric /differential thermal analysis was performed on a Netzsch STA 449F3 from 25 to 1000 °C at 5 °C/min under N_2_. The point of zero charge (pH_pzc_) analysis meant to determine the surface charge of samples prepared was carried out using the salt addition method, as described by Unuabonah et al. [22]. Elemental analysis to determine the amount of C, H, and N in the samples was performed with an Elementar Vario EL III elemental analyzer.

### Adsorption of 4-nitrophenol

For each measurement, 0.7 g of 2Z-HYCA was added to a Salamander tubular reactor (Cambridge Reactor Design Ltd, UK) operating at 30 °C. The fixed bed reactor (length of 3.625 cm, diameter of 6 mm) was flushed with 20 mL of deionized water to wash the 2Z-HYCA material. With the column delivering clean and clear deionized water and with the deionized water allowed to run out completely, a 1 mg/L solution of 4-nitrophenol was allowed to flow through the reactor bed in upward flow mode at a flow rate of 10 mL/min. Effluents from the column were collected at selected time intervals. An analysis of 4-nitrophenol was performed according to Al-Asheh et al. [25] using a 0.5 mol/L sodium carbonate solution, and measurements of the absorption of the solutions at 400 nm vs distilled water were carried out. Kyplot 2.0 software was used to model the experimental data against the Thomas model [26] by minimizing the sum of squared differences between the experimental and predicted values of the dependent variable using the quasi-Newton least squares algorithm.

### Pathogenic pollutant removal

To evaluate the efficiency of 2Z-HYCA for pathogen removal from water in a real application, commercial Eva^®^ drinking water was used for the removal experiments. Freshly purchased Eva^®^ water does not contain *E. coli* and was therefore used as the reference. *E. coli* ATCC 25922 cultures were grown in nutrient broth at 37 °C for 24 h to yield a cell count of approximately 10^9^ cfu/mL. The tip of a sterile inoculation loop was then used to spike 1 L of the water six times, which yielded approximately 10^3^ cfu/mL as measured using the optical density method that utilized a UV–vis spectrophotometer at an absorption maximum of 600 nm. This step was repeated twice, and the average optical density readings were determined.

A sample of 0.5 g of 2Z-HYCA was pretreated with 10 mL of ethanol and then dried in an oven. Subsequently, the sample was placed in an autoclave-sterilized fixed bed column (8 mm internal diameter and length 28 cm) and flushed with warm sterile water. The *E. coli*-spiked water was then passed through the bed of the 2Z-HYCA adsorbent, and effluent samples were collected at specified time intervals. To test for the presence of *E. coli* in the effluent, 1 mL of the effluent solution samples were inoculated in eosin methylene blue (EMB) agar plates, incubated at 37 °C for 24 h, and *E. coli* colonies (as indicated by a metallic sheen on the plates) were counted with a colony counter. This test was conducted in duplicate.

## Results and Discussion

### Physicochemical analysis

#### Specific surface area analysis

**Figure 1:**
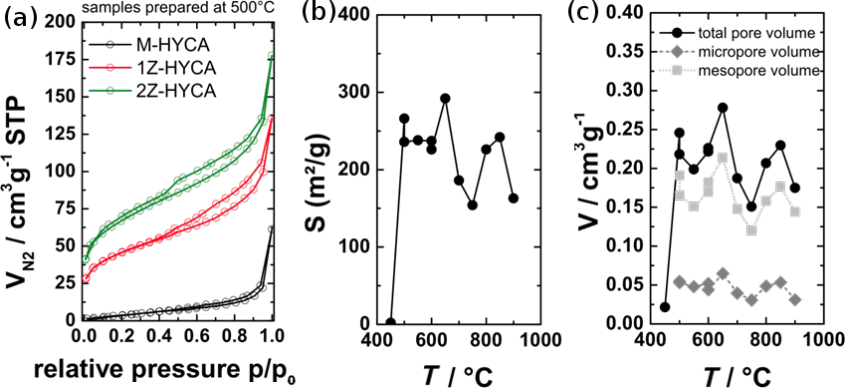
(A) N_2_ adsorption/desorption isotherms (77.4 K) for the HYCA materials prepared at 500 °C. (B) BET surface area of the 2Z-HYCA materials vs calcination temperature. (C) Pore volumes of the 2Z-HYCA nanocomposite materials vs calcination temperature.

To evaluate the effect of the type of metal salt on the efficiency of the resulting materials to remove 4-nitrophenol and *E. coli* bacteria, we prepared an initial set of materials with MgCl_2_ and ZnCl_2_ at a reaction temperature of 500 °C. Figure 1a shows the nitrogen sorption isotherms of 1M-HYCA, 1Z-HYCA, and 2Z-HYCA (see experimental part for sample labels). The shape of the isotherms suggests the presence of micro- and mesopores in 1Z- and 2Z-HYCA, while 1M-HYCA only shows a low nitrogen uptake. Its specific surface area, *S*_BET_, is only 20 m^2^/g, while the *S*_BET_ of 1Z-HYCA is 162 m^2^/g and the *S*_BET_ of 2Z-HYCA is 228 m^2^/g (Figure 1A). As a result, M-HYCA was not considered any further because high surface areas are a prerequisite for successful water treatment.

In contrast, both Zn-containing samples show adsorption–desorption isotherms indicative of porous materials. The isotherm shape is, however, rather ill-defined and can be understood as a mixture of different isotherm types. A significant uptake is observed at low relative pressure (*p*/*p*_0_), which is indicative of some microporosity. A monotonic increase in the adsorbed gas amount is observed at intermediate relative pressures, followed by a steeper volume increase at high relative pressures. The increase can be related to the condensation of N_2_ in the interstitial voids among the particles. A very modest hysteresis is observed, which closes at approximately *p*/*p*_0_ = 0.45. This result is indicative of a few mesopores with restricted access within the material, which are emptied by cavitation [27].

Furthermore, analysis of 2Z-HYCA vs the synthesis temperature indicates that significant porosity in the materials is generated at approximately 500 °C. Indeed, 2Z-HYCA prepared at 450 °C does not show any porosity, while materials prepared at 500 °C and higher show surface areas that remain roughly constant until a preparation temperature of 900 °C. No clear trend of the specific surface areas or the pore volume is observed with increasing temperature. The specific surface areas are scattered at approximately 230 m^2^/g (Figure 1b), and the total pore volume scatters at approximately 0.23 cm^3^/g (Figure 1c).

Pore size distributions (PSDs) were determined from the adsorption branches of the isotherms using a commercialized QSDFT methodology [28]. A rather constant micropore content was observed (Figure 1c) along with mesopores. The ratio of the mesopore surface area to micropore surface area is approximately 1. The pore volume of mesopores is accordingly significantly higher than the pore volume of the micropores (Table S1, Supporting Information File 1). The size distribution of the mesopores is broad (Figure S1, Supporting Information File 1) with the main fraction of pores between 2 and 8 nm. Similar to the total surface area, the PSD is independent of the synthesis temperature.

Overall, nitrogen sorption analysis indicates that the porosity does not directly correlate with the synthesis conditions as soon as the threshold temperature of 500 °C is passed.

As stated above, a high surface area is a key requirement for a material to work in water treatment. The remainder of the article will therefore focus on 2Z-HYCA nanocomposite materials which had the highest surface area.

#### Influence of synthesis temperature

**Figure 2:**
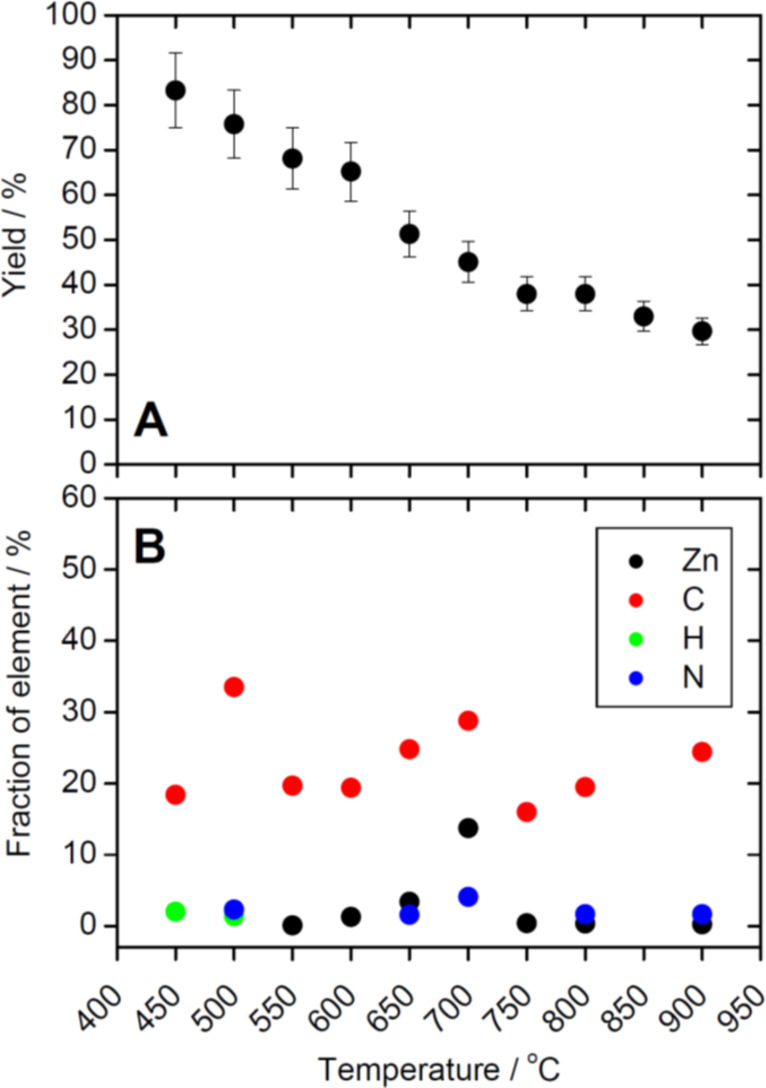
(A) Yield vs reaction temperature. (B) Chemical composition data from elemental analysis (EA) and energy dispersive X-ray spectroscopy (EDXS): Zn, C, H, and N fractions vs temperature. No symbol in (B) indicates that the concentrations of the respective elements are below the limit of detection of the respective instrument (EA = 0.3%, EDXS = 0.1%).

Figure 2A shows the isolated yields of 2Z-HYCA after the reaction at different temperatures. The data clearly show that the yield decreases as the reaction temperature increases.

In light of the need for a (reasonably) sustainable and efficient process with good yields, the reaction temperature should thus be as low as possible to provide the highest mass possible. In spite of this, the reaction temperature must be high enough to provide a high surface area and substantial transformation into the desired high surface area 2Z-HYCA nanocomposite materials. As a result, a reaction temperature of approximately 500 °C appears the most suitable for the synthesis of 2Z-HYCA nanocomposite because it is high enough to produce a material with a high surface area, yet low enough to produce substantial yields. A reaction temperature of 500 °C is even more attractive if the aspect of reduced energy cost for reactions at lower temperatures (500 vs 700 or even 900 °C) is considered. Specifically, the lower the reaction temperature is, the lower the energy cost for producing the material is.

#### Elemental analysis

The 2Z-HYCA nanocomposite samples were further analyzed with energy dispersive X-ray spectroscopy (EDXS) and elemental analysis (EA) to determine the elemental make-up of the nanocomposite and to specifically determine if it was successfully Zn doped. Figure 2B shows the fractions of Zn, C, H, and N vs sample treatment. The EA shows that increasing the reaction temperature eliminates hydrogen from the samples. This finding is consistent with previous work showing that activation using ZnCl_2_ induces the loss of hydrogen and oxygen atoms from carbon materials in the form of water, rather than as hydrocarbons or as oxygenated organic compounds [29]. The hydrogen loss could have led to aromatization of the carbon skeleton, and the formation of pores. Some authors suggest that ZnCl_2_ is very mobile above its melting point (283 °C) which inhibits the formation of volatile matter (devolatization) and thus allows for the development of a microporous structure [30–31].

The amount of Zn (obtained from EDXS) is shown to increase in 2Z-HYCA nanocomposites prepared at temperatures below 700 °C and reaches a maximum at this temperature. It then decreases sharply above 700 °C while remaining practically constant at very low levels in all samples prepared at 750 °C or higher (Figure 2B). We speculate that the tendency to incorporate Zn into the final material is maximized at temperatures of 650 and 700 °C but is greatly reduced at temperatures above the boiling point of ZnCl_2_ (732 °C) due to the evaporation of ZnCl_2_ [30].

#### Analysis of chemical functionalities

**Figure 3:**
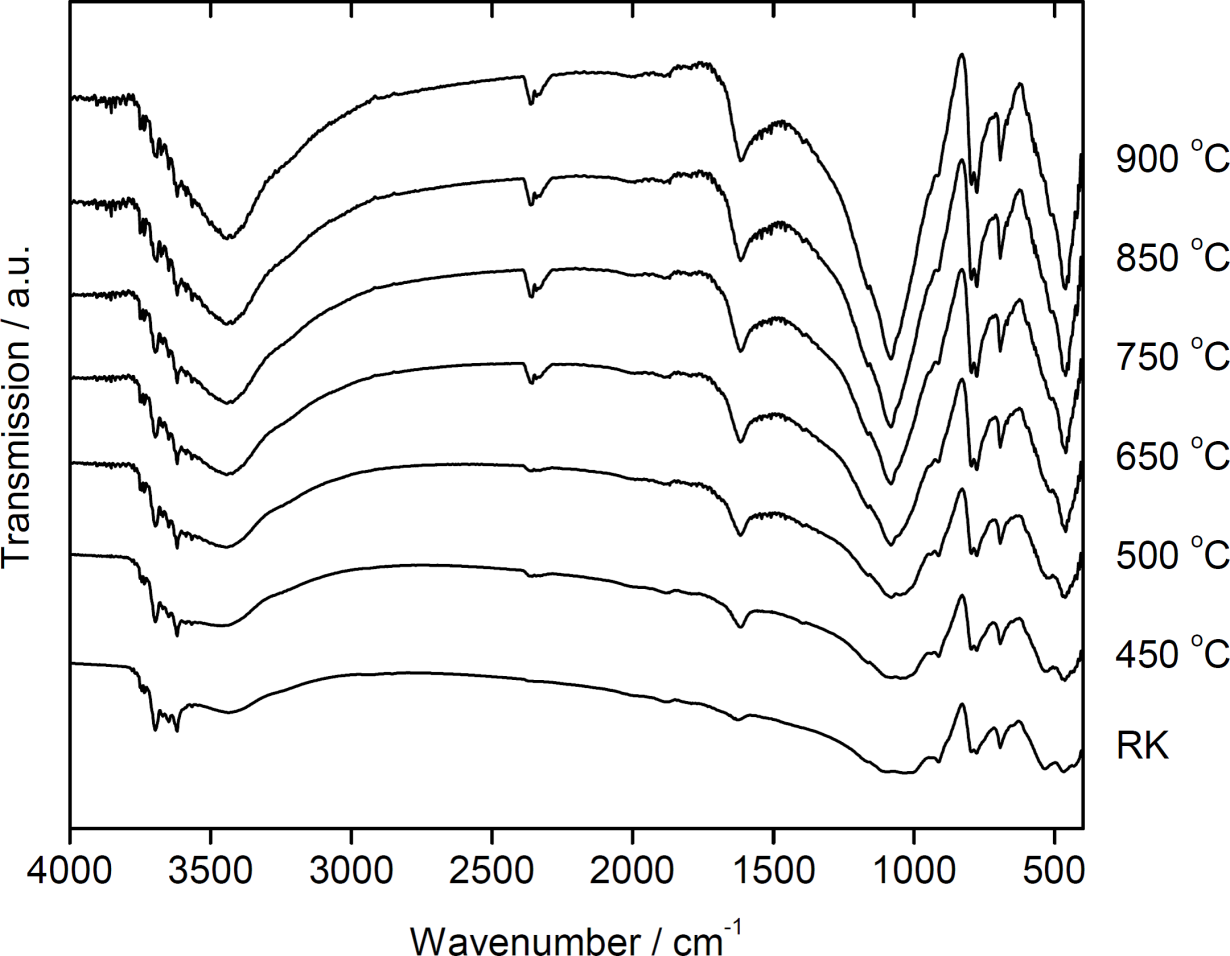
Fourier transform infrared spectra of the 2Z-HYCA nanocomposites obtained at various temperatures. “RK” indicates the spectrum of the raw kaolinite mentioned in the text.

Figure 3 shows selected FTIR spectra of 2Z-HYCA prepared at different temperatures. The signals are indicative of surface octahedral –OH groups at 3697, 3670, and 3649 cm^−1^, which are observed in the spectra of the raw kaolinite, and are no longer visible in the spectra of the 2Z-HYCA samples. This result is similar to our previous HYCA materials prepared with NaOH in air at 300 °C [1].

The broad band at approximately 3426 cm^−1^ in raw kaolinite clay is an –OH stretching vibration that is present in all 2Z-HYCA micro/mesoporous nanocomposite materials. The –OH bending vibration from adsorbed water at ≈1600 cm^−1^ decreases in intensity and shifts from 1624 to 1585 cm^−1^ with increasing reaction temperature.

The bands observed in the spectra of raw kaolinite between 1000–1200 cm^−1^ significantly change upon heating. The spectra of samples produced at 450 °C exhibit new bands at 1067 and 459 cm^−1^, which become sharper in 2Z-HYCA nanocomposite materials prepared above 450 °C. These new well-defined peaks suggest a mixed phase of Si–O and SiO_4_ tetrahedra [32]. The new distinct peak at 459 cm^−1^ is associated with the presence of Si–O [33].The –OH bending vibration from absorbed water in raw kaolinite clay (1630 cm^−1^) shifts to lower wave numbers in 2Z-HYCA (between 1615 and 1599 cm^−1^) with increasing temperature. The band at approximately 1700 cm^−1^ is attributed to the C=O stretching vibrations of carbonyl groups present in the organic fraction in the 2Z-HYCA composites. There appears to be a doublet peak at 2352 and 2356 cm^−1^ which signifies the presence of the –C–N stretching mode [34]. The Al–O absorption peak at 917 cm^−1^ decreases in intensity as the temperature increases. As a result, IR spectroscopy demonstrates that the 2Z-HYCA nanocomposite material is a complex hybrid material containing Si–O, Si–O, Al–O, –OH, –C–N, and C=O.

#### X-ray diffraction analysis

**Figure 4:**
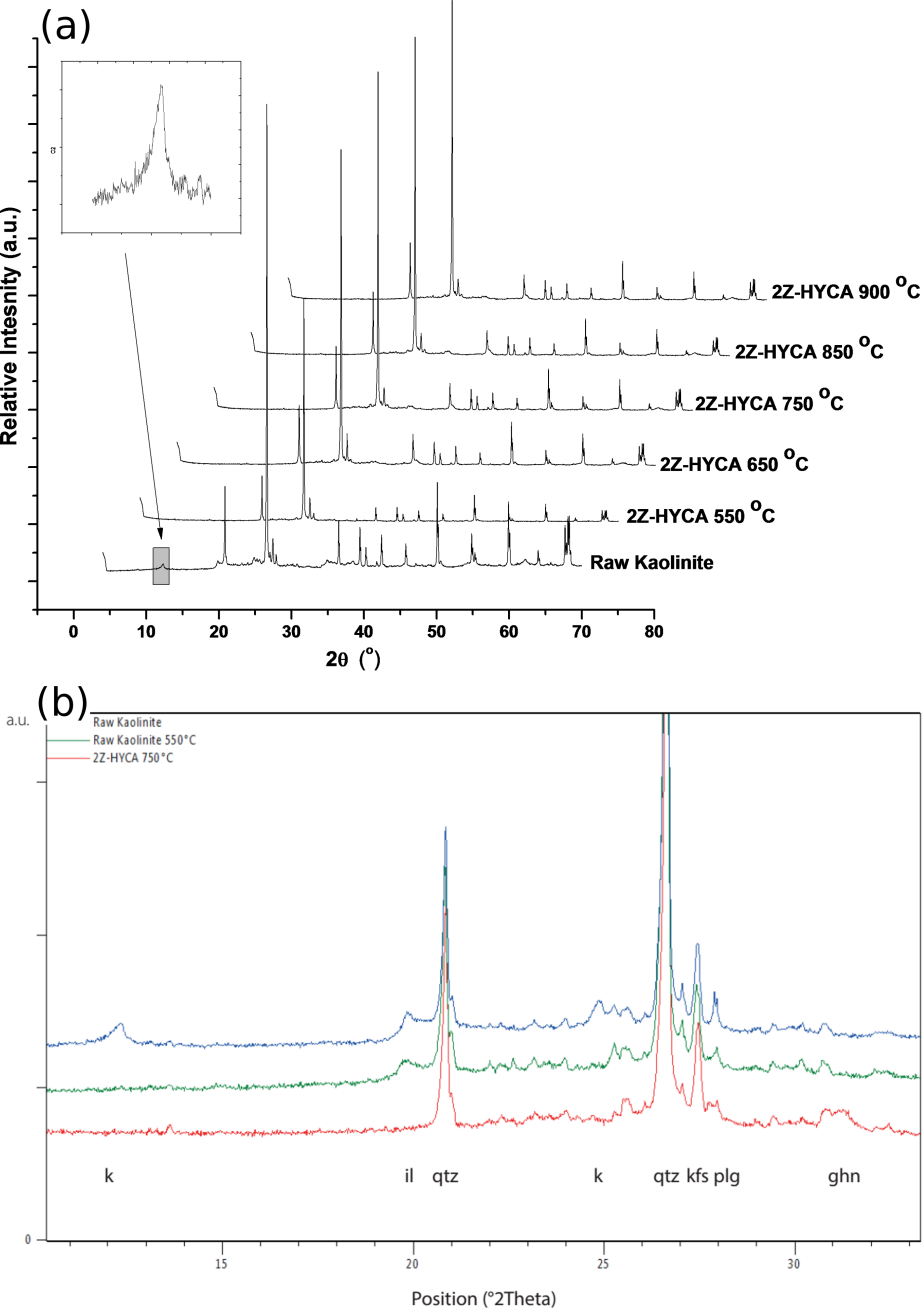
(a) XRD patterns of raw kaolinite clay and 2Z-HYCA composite materials obtained at various temperatures. (b) Powder X-ray diffraction (PXRD) diagrams of raw kaolinite (RK), raw kaolinite heated at 550 °C and 2Z-HYCA prepared at 750 °C. k = K-feldspat (microcline), il = illite, qtz = quartz, plg = plagioclase, ghn = gahnite.

Figure 4a shows the powder X-ray diffraction (PXRD) diagrams of raw kaolinite (RK) and 2Z-HYCA prepared at different temperatures. After heating, dehydroxylation of kaolinite occurs, which causes the reflections of kaolinite to disappear (for example, at 12.35° and 20.34°, 2θ, JCPDF 98-008-7771). In the absence of the reflections of kaolinite, the remaining reflections of quartz (JCPDF 98-008-9277 at 2θ values of 20.83°, 26.61°, 36.49°, 39.43°, 50.08°, etc.), K-feldspar (microcline, JCPDF 98-020-2423 at 2θ values of 21.04°, 27.07° and 27.45°) and plagioclase (JCPDF 98-003-4917 at 2θ values of 22.01° and 27.95°) occur more clearly.

Figure 4b shows the comparison of the PXRD diagrams of raw kaolinite, raw kaolinite heated at 550 °C and 2Z-HYCA heated at 750 °C for one hour. The originally used raw kaolinite is composed of kaolinite (k), quartz (qtz) and feldspar (microcline (kfs) and plagioclase (plg)) and small amounts of illite (il, JCPDF 98-009-0144) and anatase (TiO_2_, JCPDF 98-009-6946, at 2θ values of 25.29°). As already mentioned, the kaolinite reflections disappear for all heated samples. Additionally, the illite reflection (see Figure 4b at 2θ 19.82°) decreases, probably due to the dehydroxylation illite, which begins at temperatures >550 °C. Additionally, above 650 °C, the heated 2Z-HYCA diagrams show broad reflections (see Figure 4a at 2θ values of 31.25, 36.82°, 55.62°, 59.32° and 65.19°), which can be assigned to a new Al_2_ZnO_4_-phase (ghn, gahnite-type JCPDF 98-007-5098).

Interestingly, the new materials prepared in this study exhibit no ZnO phase, as seen from the FTIR or XRD spectra, unlike in our previous studies [21]. This behavior is because a standard solution of NaOH used in the previous preparation of the materials (which, when reacted with ZnCl_2,_ will yield ZnO) was omitted in the preparation of the nanocomposites in this study.

#### Nuclear magnetic resonance spectroscopy analysis

**Figure 5:**
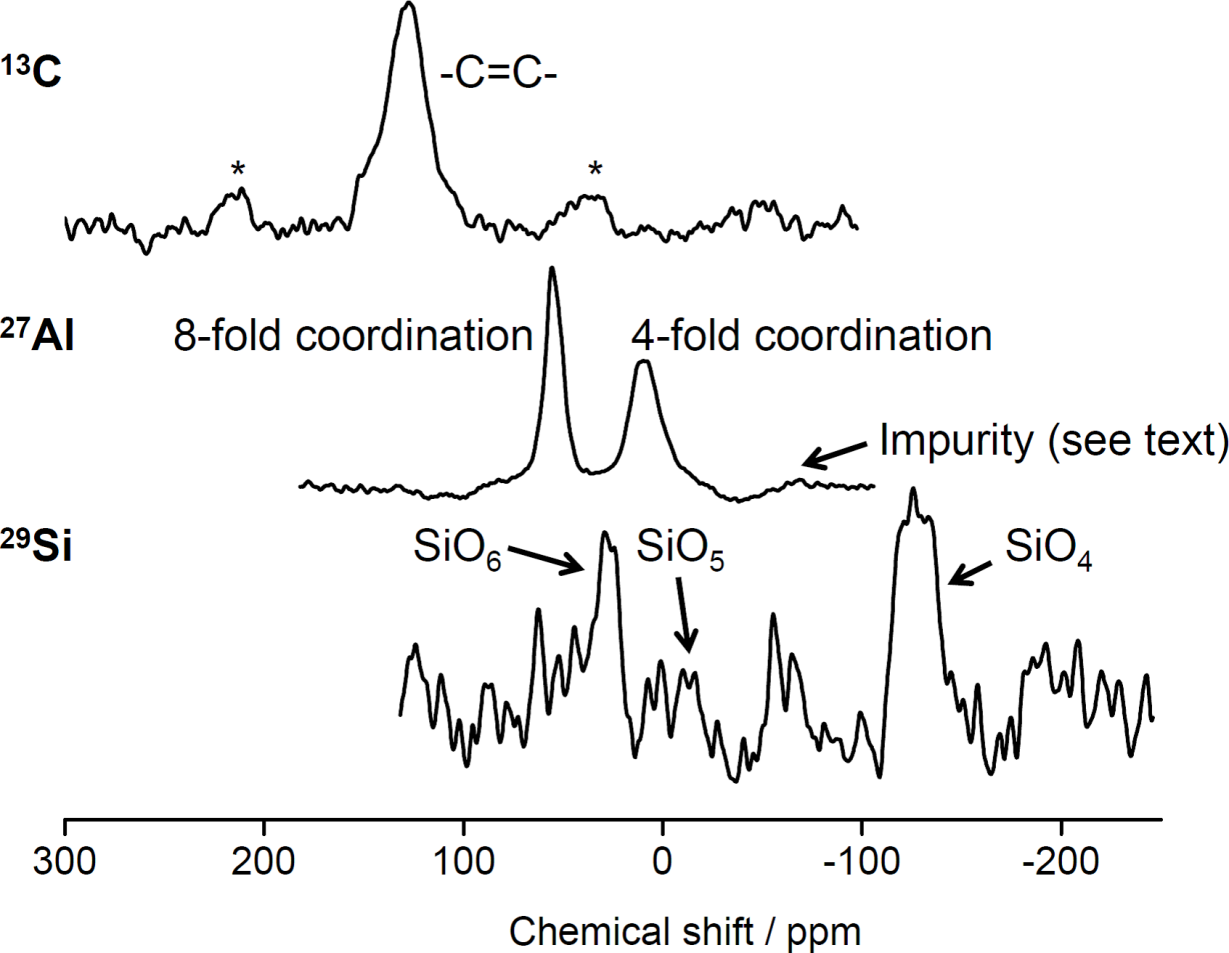
^29^Si, ^27^Al and ^13^C CP-MAS-NMR spectra of 2Z-HYCA calcined at 650 °C. *Rotation side bands of ethylene carbon.

To further evaluate the composition and structure of 2Z-HYCA, a sample calcined at 650 °C was investigated with solid-state cross-polarized magic angle spinning nuclear magnetic resonance (CP-MAS-NMR) spectroscopy. The corresponding ^29^Si, ^27^Al, and ^13^C CP-MAS-NMR spectra are shown in Figure 5.

The ^13^C spectrum only shows a prominent peak at 128 ppm, indicating the presence of sp^2^-bonded carbon environments, specifically CH=CH_2_ moieties [35]. The weak bands at ≈215 and ≈35 ppm are rotation sidebands [36].

The ^29^Si MAS-NMR spectrum shows a set of low-resolution signals. The bands at −89, −100 and −126 ppm correspond to Q^3^ (1Al) [37], isolated silanol [SiO_3_(OH)] [38] and Q^4^ (0Al) [37] silicon sites of metakaolin, respectively. These peaks are common to kaolinite heated between 550–850 °C [38]. The presence of these peaks suggests that 2Z-HYCA is an organic–inorganic hybrid material.

The ^27^Al MAS-NMR spectrum shows two distinct peaks at 55 and 10 ppm, characteristic of tetrahedrally (T) and octahedrally (O) coordinated aluminum centers, respectively, in metakaolin [39–40]. The T/O ratio suggests that the fraction of T-coordinated Al is almost twice that of O-coordinated Al. A broader resonance with low intensity is observed at ≈−68 ppm, perhaps resulting from an amorphous or less crystalline (impurity) phase [41].

Overall the XRD and solid-state NMR data support the FTIR spectroscopy spectra in that all methods detect some chemical changes in the zinc-based HYCA materials compared to the starting materials, thus confirming the formation of a real hybrid material. This result is also confirmed by SEM.

#### Scanning electron microscopy analysis

**Figure 6:**
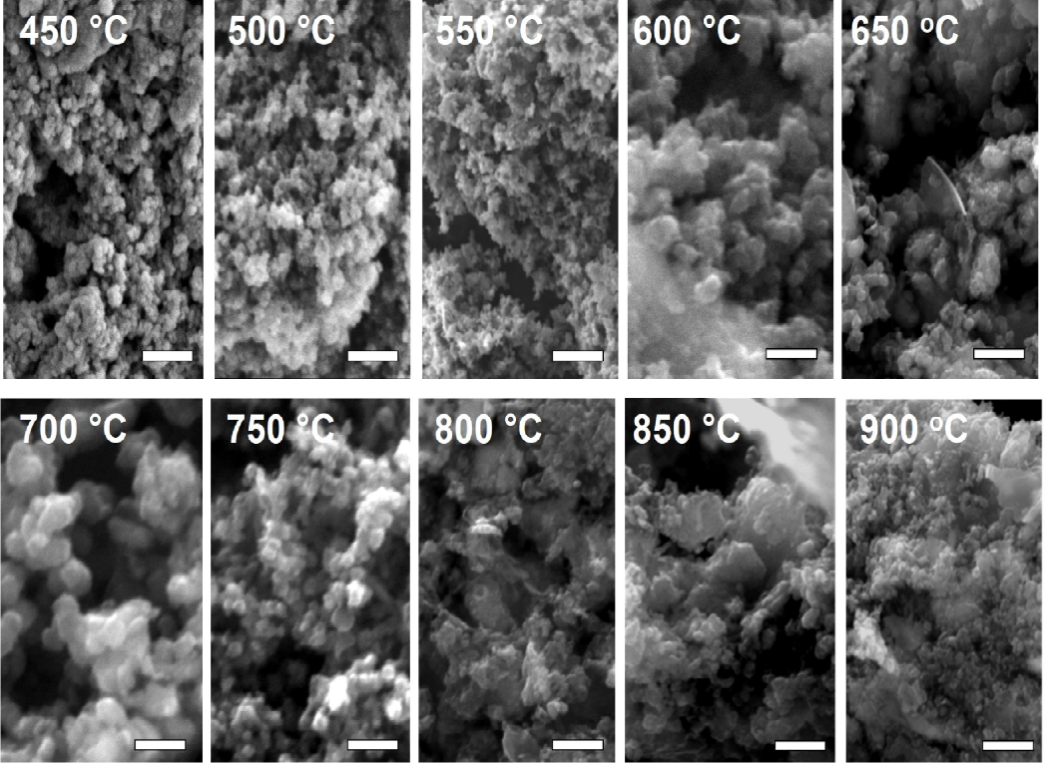
SEM images showing the growth pattern of 2Z-HYCA nanocomposite particles with increasing temperature after acid washing. All scale bars = 1 μm.

Figure 6 shows representative SEM images of all samples. Although the morphologies of the materials synthesized at different temperatures are quite similar, some differences can be observed. In all cases, roughly spherical primary particles are observed, but the materials produced at lower temperatures appear to consist of smaller particles (approximate diameter between ≈50 and 150 nm), while the samples produced at higher temperatures contain larger particles with approximate diameters of 200 to 300 nm. Moreover, the samples made at 750 °C and higher also contain plate-like or fiber-like features.

#### Thermogravimetric and differential thermal analysis

**Figure 7:**
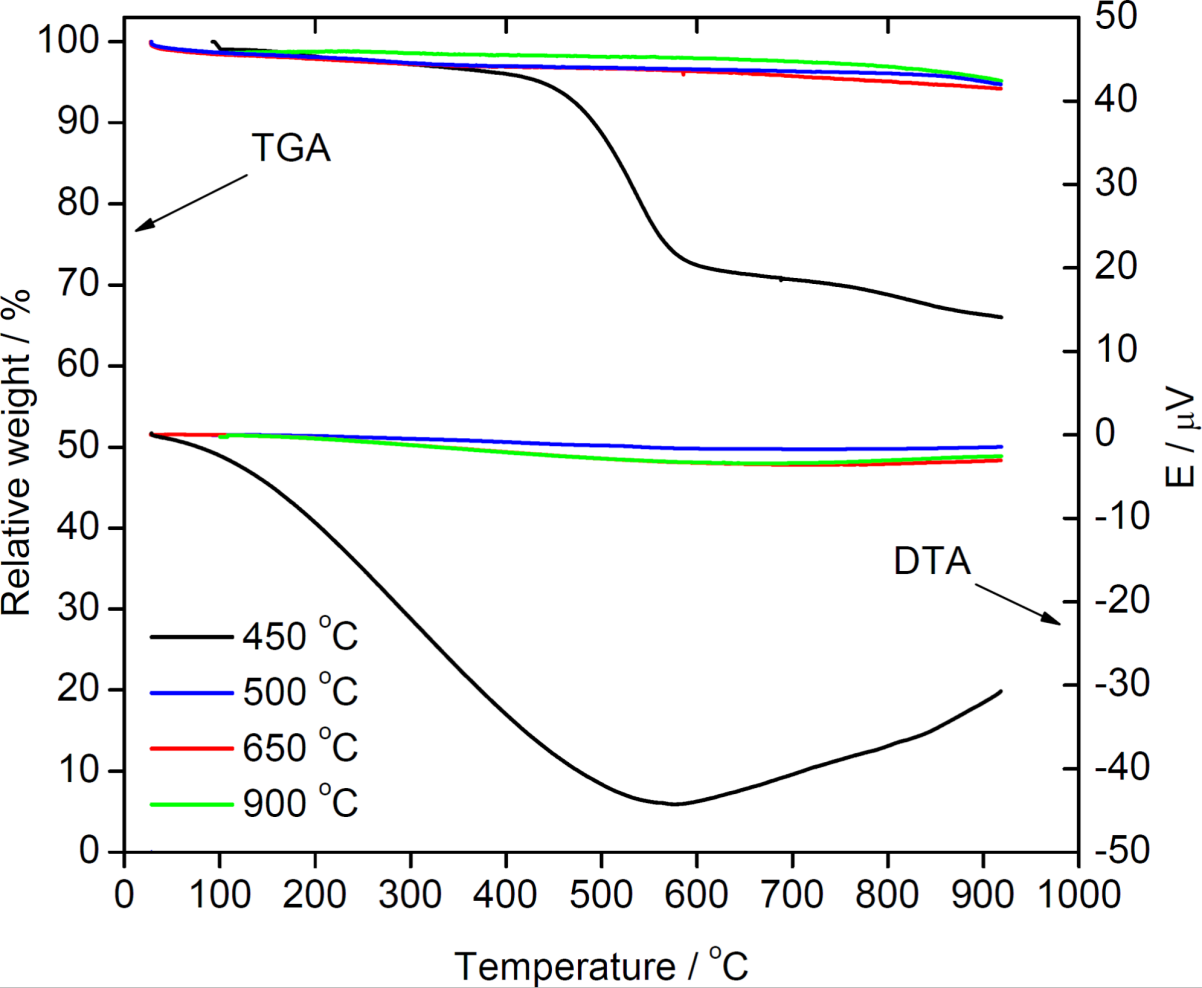
TGA and DTA curves for 2Z-HYCA nanocomposites prepared at various temperatures.

Figure 7 shows representative thermogravimetric analysis/differential thermal analysis (TGA/DTA) data obtained from measurements in nitrogen. Consistent with the data shown above, there are clear differences between samples produced at reaction temperatures up to 450 °C and the samples obtained at higher temperatures. The samples made between 500 and 900 °C only show a weak and very gradual weight loss of ≈5–6% of the total mass at the end of the TGA/DTA experiment. This finding indicates that most of the volatiles have already been eliminated during the synthesis of the materials.

In contrast, the samples produced at lower temperatures are much less stable and lose up to 45% of their original mass. Here, the weight losses can be separated in three (although strongly overlapping) steps between 25 and 100 °C, 110 and ≈450 °C, and finally ≈450 to 900 °C [42]. The first loss can be assigned to the desorption of water adsorbed in the pores of the hybrid clay materials. The second loss is attributed to the pre-dehydration process as a result of a reorganization in the octahedral layers in kaolinite associated with condensation and water elimination [43–44]. The third loss is assigned to the dehydroxylation of kaolinite (and formation of metakaolinite), which is supported by an exothermic peak in the thermogravimetric analysis (DTA) [42]. This explains the loss of the inner hydroxyl related to kaolinites initially observed from our FTIR analysis in this study. Moreover, some fraction of the weight losses at higher temperatures may also be assigned to condensation reactions in the inorganic components and concurrent water elimination, but these individual processes cannot be separated here.

TGA/DTA data analysis therefore indicates that the reaction temperature is indeed a key parameter to obtain a stable material that will resist rapid attrition during use. Comparison with the nitrogen sorption data suggests that a reaction temperature of ≈500 °C is interesting both from stability and surface area aspects.

### Pollutant removal from aqueous solutions

#### Removal of 4-nitrophenol

We have previously reported that a first-generation HYCA material prepared via a low-temperature calcination process in air very efficiently adsorbs cationic pollutants such as Pb^2+^, Cd^2+^, Ni^2+^ [1], and methylene blue [4] from an aqueous solution. However, this material fails when attempting to remove anionic pollutants such as 4-nitrophenol (with a p*K*_a_ of 7.2 [45]), methyl orange dye, or phosphate.

The data demonstrate that the 2Z-HYCA nanocomposite is able to overcome the limitations of the highly negatively charged original HYCA material [1] and provides sites for the adsorption of anionic pollutants, such as 4-nitrophenol.While used as a model compound here, 4-nitrophenol is an organic anionic pollutant of high interest because acute exposure to 4-nitrophenol causes blood disorders or liver and kidney damage [46–47].

**Figure 8:**
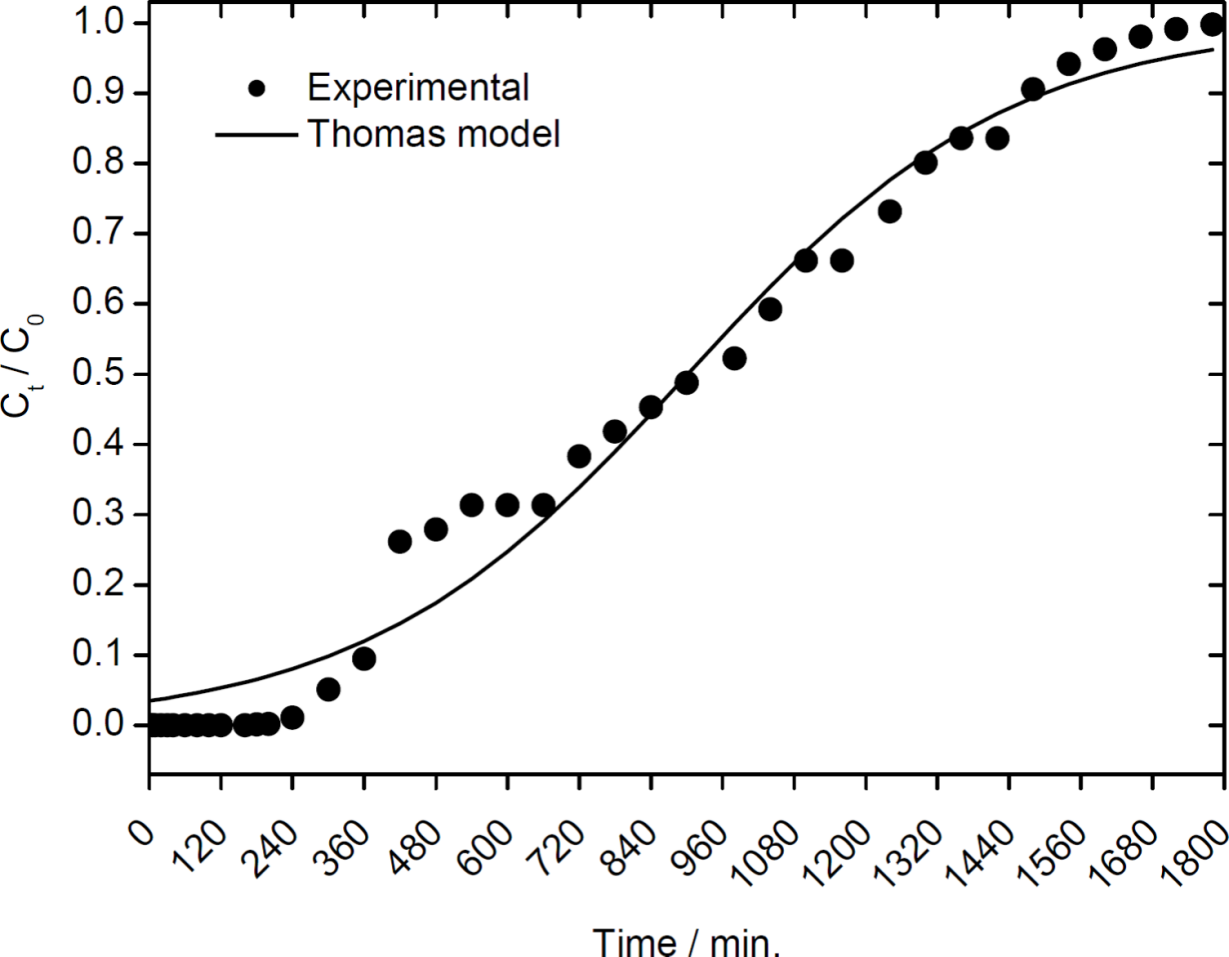
Experimental breakthrough curve for the adsorption of 4-nitrophenol onto 2Z-HYCA@650 °C (*C*_o_ = Concentration of 4-nitrophenol at time 0; *C*_t_ = Concentration of 4-nitrophenol at time *t*).

Figure 8 shows a representative dataset obtained from a setup with 0.7 g of 2Z-HYCA@650 °C (the material with the highest surface area) in a fixed bed reactor. 2Z-HYCA@650 °C reduces the concentration of 4-nitrophenol to below the detection limit after 80 min when aqueous solutions with 4-nitrophenol concentrations of 1 mg/L were used. Moreover, a 50% breakthrough was reached at ≈900 min after treating 9 L of the same solution, and the 2Z-HYCA@650 °C was 100% spent after 28 h 40 min. This result demonstrates that 2Z-HYCA is a highly effective adsorbent for 4-nitrophenol, especially when considering the very short empty bed contact time (the time a treated solution is in contact with 2Z-HYCA@650 °C nanocomposite material) of 8.6 s, as determined from the AdDesignS^TM^ software [48]. The rate constant obtained by fitting the data with the Thomas model (Figure 8) is 3.69 mL/min·g, and the adsorption capacity is 3.61 mg/g.

The data obtained were modelled against the pore and surface diffusion model (PSDM) and the constant pattern homogeneous surface diffusion model (CPHSDM) using the AdDesignS^TM^ software and making various inputs (parameters given in Table 1).

**Table 1:** Input data for pore and surface diffusion model (PSDM) and constant pattern homogeneous surface diffusion model (CPHSDM) modelling using AdDesignS software.

**Input data for CPHSDM**	

**chemical**	
molecular weight of 4-NP	139 g/moL
initial concentration	1 mg/L
**bed data**	
bed length	5.075 × 10^−2^ m
bed diameter	6.000 × 10^−3^ m
weight of HYCA	0.7 g
inlet flow rate	10 mL/min
temperature	30 °C
water density	30 °C = 0.9957 g/cm^3^
water viscosity	8.15 × 10^−3^ g/cm·s

**input data PSDM**	

**chemical**	
molecular weight of 4-NP	139 g/moL
initial concentration	1 mg/L
**bed data**	
bed length	5.075 × 10^−2^ m
bed diameter	6.000 × 10^−3^ m
weight of HYCA	0.7 g
inlet flow rate	10 mL/min
temperature	30 °C
water density	30 °C = 0.9957 g/cm^3^
**adsorbent properties**	
name	2Z-HYCA
apparent density	2.42 g/cm^3^
particle radius	0.030000 cm
porosity	1.000

**Figure 9:**
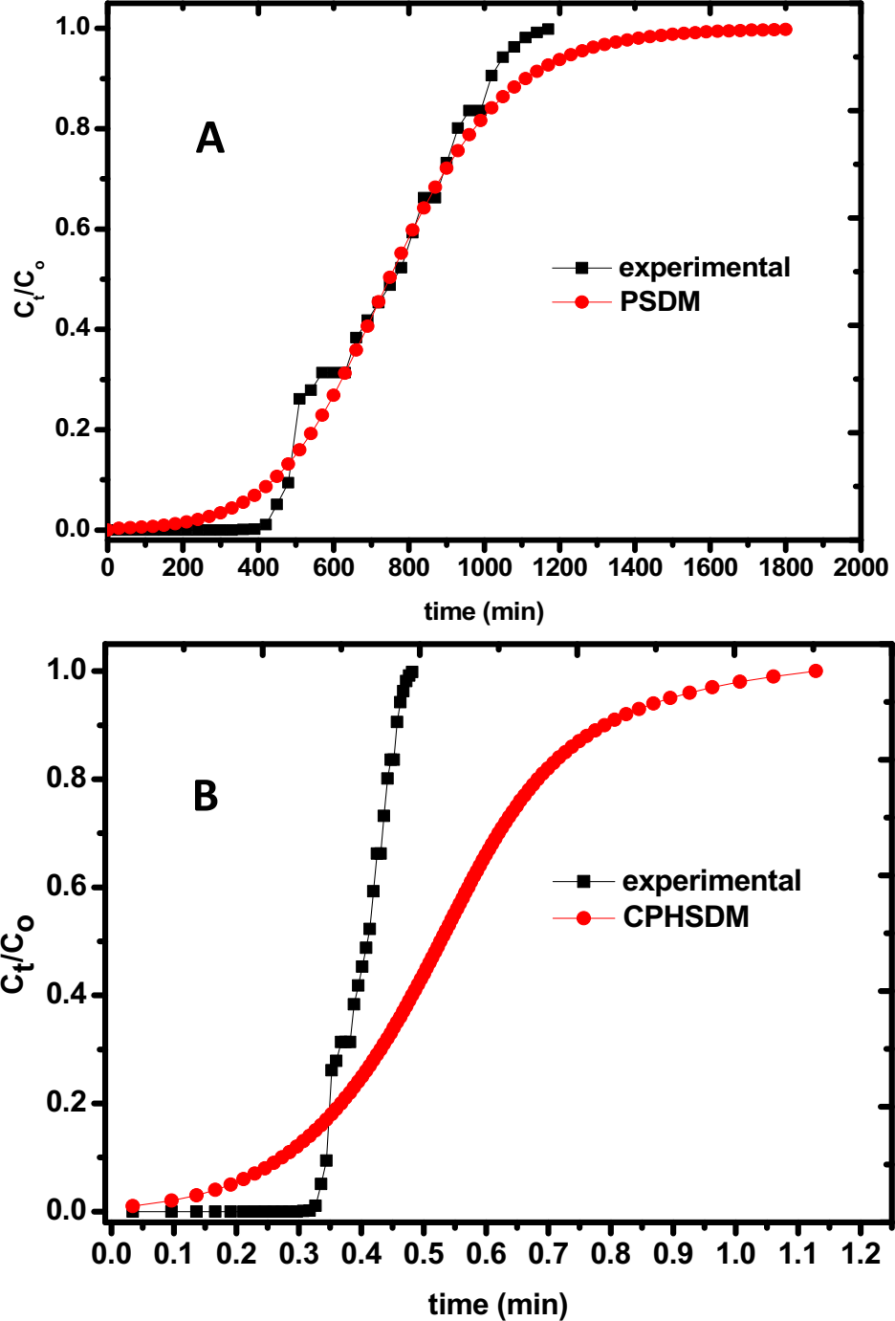
(A) Pore and surface diffusion model (PSDM) plot for the removal of 4-nitrophenol from aqueous solution by 2Z-HYCA@650 °C loaded into a fixed-bed reactor (*C*_o_ = initial concentration of 4-nitrophenol at time 0 and *C*_t_ = concentration of 4-nitrophenol at time *t*). (B): Constant pattern homogeneous surface diffusion model (CPHSDM) plot for the removal of 4-nitrophenol from aqueous solution by 2Z-HYCA@650 °C loaded into a fixed-bed reactor (*C*_o_ = initial concentration of 4-nitrophenol at time 0 and *C*_t_ = concentration of 4-nitrophenol at time *t*).

The results suggest that data obtained for the adsorption of 4-nitrophenol onto 2Z-HYCA@650 °C nanocomposite is mainly by a pore and diffusion mechanism as supported by the good fit to PSDM (Figure 9A) rather than a surface reaction that is supported by the CPHSDM that showed a very poor fit with the data (Figure 9B).

The PSDM predicted that 1 kg of 2Z-HYCA@650 °C micro/mesoporous nanocomposite material can reduce a concentration of 1 mg/L of 4-nitrophenol (single solute solution) in 290 L of aqueous solution below 50 μg/L in 346 min (5 h 46 min). To maintain this same concentration of 4-nitrophenol in aqueous solution below the World Health Organization’s drinking water equivalent level (DWEL) of 0.3 mg/L [49], 1 kg of 2Z-HYCA@650 °C will be required for ≈10 h 20 min and will treat 2.49 m^3^ of water per day.

#### Removal of *Escherichia coli*

**Figure 10:**
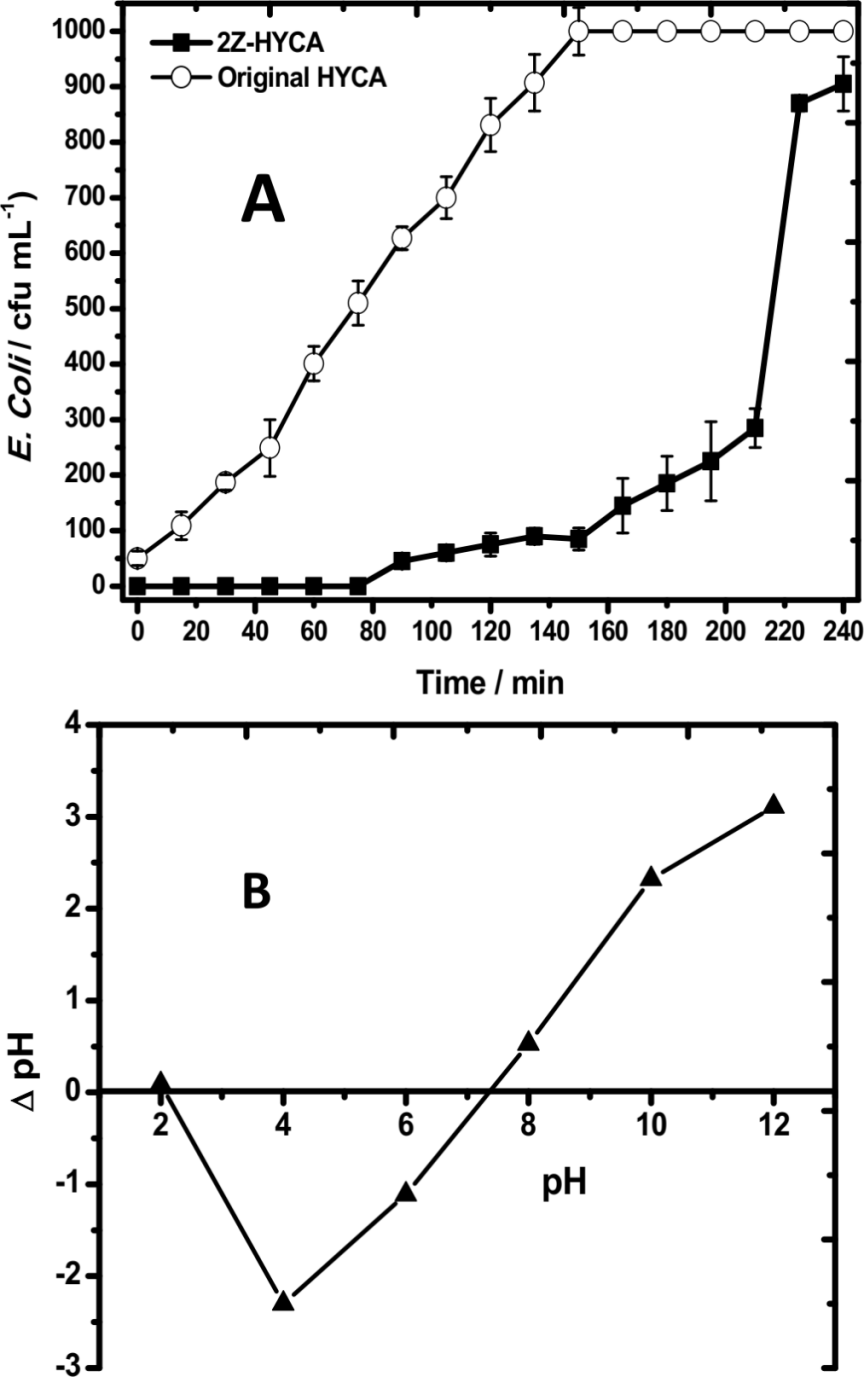
(A) Bacterial load (*E. coli*) vs treatment time measured in water treated with a fixed bed process for both the original HYCA material and the new 2Z-HYCA@650 °C material. The initial *E. coli* concentration was 10^3^ cfu/mL; (B): The pH_pzc_ plot for 2Z-HYCA@650 °C.

Figure 10A shows that 0.5 g of 2Z-HYCA@650 °C essentially eliminates *Escherichia coli* (*E. coli*) completely from water, which was initially spiked with *E. coli* (at 10^3^ cfu/mL), within 75 min.

After 75 min, the amount of *E. coli* in the treated water increased with time. In contrast, the regular HYCA material [1] can also remove *E. coli* from the same solutions but with a much lower efficiency. This finding suggests that the presence of Zn^2+^ in the composite material directly affects *E. coli* removal from solution, likely because zinc has antibacterial properties. Although the precise functional mechanism is unknown, it has been suggested that Zn-doped materials deactivate bacteria by damaging its cell membrane and DNA [50]. It is known that electrostatic adsorbent–adsorbate interactions do occur in solution at pH values either above or below the pH_pzc_ of the adsorbent [51]. Based on the latter, it is believed that the composite adsorbent material in this study does become positively charged below its pH_pzc_ of 7.4 (Figure 10B) in an aqueous mixture of bacteria (whose pH was measured to be 6.36) since a mixture of bacteria solution and nanocomposite will reduce the pH to below 7.4. This will aid electrostatic interaction between the negatively charged bacteria and the positively charged 2Z-HYCA@650 °C. This behavior also explains, in part, the mechanism for the uptake of 4-NP by 2Z-HYCA nanocomposite material, as it is expected that 4-nitrophenol will ionize in water at pH 6.3 to yield some 4-nitrophenoxide anions that will be electrostatically held onto the positive sites on the surface of the material.

Even after 3 h of run time, the level of *E. coli* in the treated solution was still below the alert/action levels of 500 cfu/mL in drinking water for *E. coli* [52], as shown in Figure 10. This suggests the potential of the 2Z-HYCA@650 °C as a water disinfectant for the future. However, more studies still need to be conducted to ascertain this.

## Conclusion

This study introduces the newest generation of the HYCA material, namely, 2Z-HYCA, two low-cost precursor sources, kaolinite clay and *Carica papaya* seeds, synthesized without the need for alkali activation. The preparation is simple, and the resulting nanocomposite material is micro/mesoporous, unlike the initial hybrid clay material prepared in our previous study [1]. The new micro/mesoporous material is efficient for the removal of 4-nitrophenol and *E. coli* from drinking water. Zinc may not be desirable from a heavy metal pollution point of view; however, the introduction of zinc significantly enhances the performance of the original HYCA materials for remediation of these pollutants in water. As such, and because the Zinc concentration leached into treated water from the 2Z-HYCA nanocomposite material is very low [22], it is thus the belief of the authors that the dual functionality of this new material in efficiently removing a recalcitrant anionic organic pollutant (4-nitrophenol) and bacteria (*E. coli*) from water compensates for the cost of N_2_ and high temperature employed in its preparation, even in developing countries. As a result, the current approach opens a new door towards cheap and sustainable materials development with exciting performance in one of the key areas of today’s world, the treatment of water in developing countries.

## Supporting Information

The Supporting Information contains additional information on the specific surface area analysis using the quenched solid density functional theory, the raw data for elemental and specific surface area analyses.

File 1 Additional experimental results.
