# Supplementary material for: New micro/mesoporous nanocomposite material from low-cost sources for the efficient removal of aromatic and pathogenic pollutants from water
Source: Beilstein J Nanotechnol. 2019 Jan 9;10:119–31. doi: 10.3762/bjnano.10.11 (PMC6334806; doi:10.3762/bjnano.10.11)
Supplement: File 1 — Additional experimental results. [file Beilstein_J_Nanotechnol-10-119-s001.pdf]

BEILSTEIN JOURNAL OF NANOTECHNOLOGY

Supporting Information

for

New micro/mesoporous nanocomposite material from low-cost sources for the efficient removal of aromatic and pathogenic pollutants from water

Emmanuel I. Unuabonah, Robert Nöske, Jens Weber, Christina Günter and Andreas Taubert

Beilstein J. Nanotechnol. **2019,** *10,* 119–131. doi:10.3762/bjnano.10.11

Additional experimental results

License and Terms: This is a supporting information file under the terms of the Creative Commons Attribution License (http://creativecommons.org/licenses/by/4.0). Please note that the reuse, redistribution and reproduction in particular requires that the authors and source are credited.
The license is subject to the *Beilstein Journal of Nanotechnology* terms and conditions: (https://www.beilstein-journals.org/bjnano)

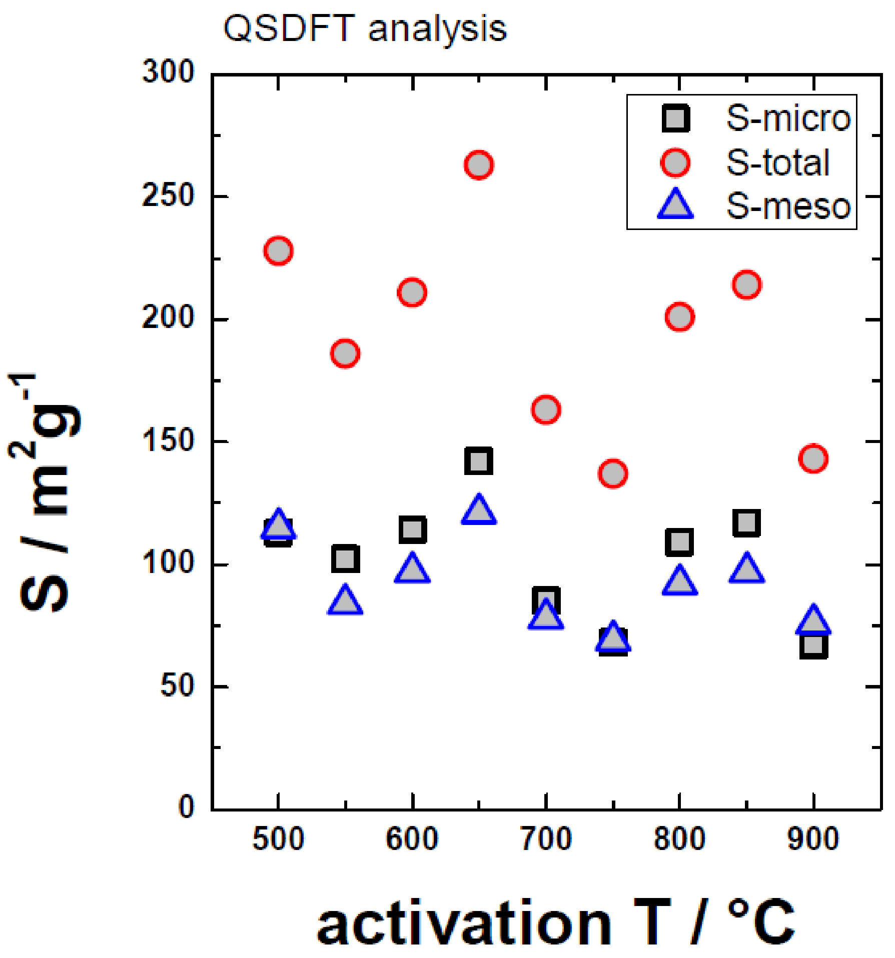

Figure S1: Specific BET surface area of various porosities in 2Z-HYCA materials vs temperature.

Table S1: Data from Elemental, BET and EDX Analyses.

	% Element				Pore Volume (cm^3/g)			Micro/Mesopore Area		
	C	H	N	BET (m^2/g)	V_{mic}	V_{meso}	$V_{mic + meso}$	SA_{mic}	SA_{mes}	SA_{mes}/SA_{mic}
$2ZHYCA_{450}$	18.4	2.0	-	2.0	-	-	0.021	-	-	2.0
$2ZHYCA_{500}$	33.5	1.4	2.3	228	0.055	0.191	0.246	113	115	1.02
$2ZHYCA_{550}$	19.7	-	-	186	0.048	0.151	0.199	102	84	0.82
$2ZHYCA_{600}$	19.4	-	-	211	0.052	0.170	0.222	114	97	0.85
$2ZHYCA_{650}$	24.8	-	1.6	263	0.065	0.213	0.278	142	121	0.85
$2ZHYCA_{700}$	28.8	-	4.1	163	0.039	0.148	0.187	85	78	0.92
$2ZHYCA_{750}$	16.0	-	-	137	0.031	0.120	0.151	68	69	1.01
$2ZHYCA_{800}$	19.5	-	1.7	201	0.049	0.158	0.207	109	92	0.84
$2ZHYCA_{850}$	24.4	-	1.7	214	0.053	0.176	0.229	117	97	0.83
$2ZHYCA_{900}$	17.1	-	1.3	143	0.031	0.144	0.175	67	76	1.13
